# Spatially Multiplexing of Metasurface for Manipulating the Focused Trefoil and Cinquefoil Vector Light Field

**DOI:** 10.3390/nano11040858

**Published:** 2021-03-27

**Authors:** Rui Sun, Chuanfu Cheng, Ruirui Zhang, Xiangyu Zeng, Yu Zhang, Manna Gu, Chunxiang Liu, Hong Ma, Qian Kong, Chen Cheng

**Affiliations:** College of Physics and Electronics, Shandong Normal University, Jinan 250014, China; sunrui199812@163.com (R.S.); zhangruirui0268@163.com (R.Z.); zengxiangyu0611@163.com (X.Z.); zhangyu_sdnu@163.com (Y.Z.); gumanna1996@outlook.com (M.G.); liuchunxiang@sdnu.edu.cn (C.L.); mahong@sdnu.edu.cn (H.M.); kongqian0304@163.com (Q.K.)

**Keywords:** metasurfaces, vector beam, polarization, vortex beam, geometric phase

## Abstract

The trefoil and cinquefoil vector field are of essential significance for fundamental topology properties as the Hopf link and trefoil knots in the light field. The spatially multiplexing metasurfaces were designed with two sets of periodical nanoslits arranged alternately, each had independent geometric spiral phases and metalens phases to produce and focus vortex of the corresponding circular polarized (CP) light. By arranging the orientations of the two slit sets, the two CP vortices of the desired topological charges were obtained, the superposition of the vortices were realized to generate the vector field. With the topological charges of the vortices set to one and two, and three and two, respectively, the focused trefoil and cinquefoil vector light fields were acquired. The work would be important in broadening the applications of metasurface in areas as vector beam generations and topology of light field.

## 1. Introduction

In recent years, vector beams with spatially non-uniform polarization states have attracted widespread attention due to their unique properties. They have shown broad application prospects in many fields such as quantum communication [1], optical tweezers [2], high-resolution microscopy [3], laser processing and structuring [4,5]. Giovanni Milione et al. systematically proposed a higher-order Poincaré sphere (HOPS) to describe a vector beam as the superposition of two symmetrical vortices of opposite circular polarizations (CPs) [6]. Later, Xunong Yi et al. extended HOPS to the hybrid-order Poincaré sphere (HyOPS) which was used to describe the more general polarization state of a vector beam formed by the superposition of two arbitrary vortices of the opposite CPs [7]. One of the unusual features of vector fields that has attracted particular interest recently is optical polarization knots and links in the complex topological configurations of the fields. The singularity phase lines along the amplitude zero will be generated, when multiple waves are superimposed. These singularity phase lines often form self-winding looped three-dimensional structures which may affect the topology of other structures in the wave field [8]. Exemplarily, when the longitudinal field component focused beams in the light field is considered, a three-dimensional topology of Möbius strip is observed [9]. Specifically, the trefoil and cinquefoil light fields are of essential significance in generation of the Hopf link and trefoil knots, demonstrating the promise of vector beams on HyOPS in the interesting research areas of light field topologies. In addition, the trefoil and cinquefoil vector fields also have potential applications in measurement of the traveled distance [10], ultrafast media patterning and nanomanipulation [11]. In the last years, various methods have been developed to generate vector beams using different optical devices, including electrically tunable liquid crystal q-plates and wave plates [12], and spatial light modulators [13].

As a planar two-dimensional metamaterial, metasurfaces are composed of subwavelength scale nanostructural units, which are ultra-thin, easy to manufacture and easy to integrate. Metasurfaces have been changing, essentially, the fundamentals and techniques of light field manipulations owing to their ability to control the amplitude, phase and polarization of the field simultaneously. At present, various metallic and dielectric metasurfaces have been designed to implement multifunctional planar optical devices, such as metalens [14,15], vortex beam converter [16] and polarization conversion devices [17,18]. Some subtle metasurfaces have been proposed to construct nanoscale topological light fields [19,20], and a metasurface that consists of rectangular gold nanorods was proposed to realize holographic reconstruction of vortex knots and links [21,22]. Guo et al. proposed and experimentally demonstrated a full-dielectric metasurface that can construct vortex knots and links at the nanoscale. The two different topological configurations can be switched by changing the polarization state of the incident light [23]. The ability of metasurfaces to change the polarization of incident light has significant advantages in constructing polarization-switchable topological configuration. In recent years, composite metasurfaces have been designed to manipulate multiple light fields independently to generate vector light fields.

In this paper, we designed a spatially multiplexing metasurface to generate focused trefoil and cinquefoil vector fields with arbitrary polarization states. By controlling the geometric phases with the varied orientations of the multiplexing slits in the metasurface, the multiplexed wave fields contained in the output field were independently manipulated. The focused vector fields were produced by superposing two CP beams of opposite helicity with different topological charges. Intuitively, the trefoil vector field was achieved by superposing the vortex beams of right circular polarization (RCP) with topological charge *l_A_* = 1 and left circular polarization (LCP) with *l_B_* = 2, and the cinquefoil vector field by superposing the vortex beams of RCP with *l_A_* = 3 and LCP with *l_B_* = 2. By simply changing signs of the topological charges *l_A_* and *l_B_*, superpositions produce the corresponding trefoil and cinquefoil π-vector fields, respectively. Based on the above principles, spatially multiplexing metasurfaces etched on gold films were designed under linearly polarized (LP) illumination containing equal LCP and RCP light. The metasurfaces were consisted of two sets of periodical nanoslits arranged alternately in the *y*-direction; the two sets of nanoslits had independent geometric phase distributions, one of the sets had the geometric spiral phase and metalens phase to produce and focus vortex of RCP, and the other set had the geometric spiral phase and metalens phase to produce and focus vortex of LCP. By designing the orientation distributions of the two slit sets in a metasurface based on the desired topological charges *l_A_* and *l_B_* for the multiplexed fields, the trefoil and cinquefoil vector fields were obtained by superposing multiplexed vortex fields with the spatially multiplexing metasurfaces. The results and generation methods are of great significance for exploring novel topological properties of light fields and for broadening the applications of the topological light fields.

## 2. Materials and Methods

**Figure 1:**
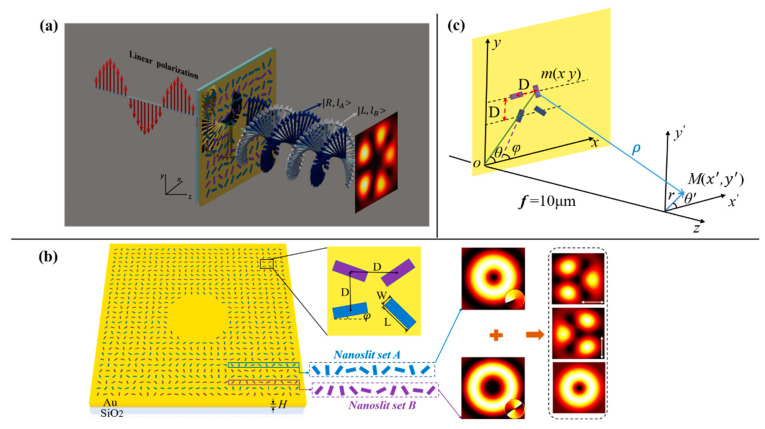
(**a**) schematic of generation of vector light field by illumination of linearly polarized (LP) light. (**b**) schematic of the metasurface and detailed view of a single slit: The length (L) and width (W) of a single slit are 250 nm and 80 nm, respectively; a slit-to-slit spacing D in both *x*-and *y*-directions; the thickness of the gold film is H = 200 nm; and the nanoslit set A and set B are arranged alternatively in the *y*-direction generate two vortices |*R, l_A_*> and |*L, l_B_*> of right circular polarization (RCP) and left circular polarization (LCP) with the topological charges *l_A_* and *l_B_*, respectively, the superposition of two focused vector vortices can produce the vector fields. (**c**) sketch map of optical geometry for light propagations and light field generation.

The schematic diagram of the spatially multiplexing of metasurface to generate converged trefoil and cinquefoil vector field is shown in Figure 1a. The metasurface consisted of two sets of nanoslits, i.e., nanoslit set A and set B, which produced two CP vortices |*R, l_A_*> = *e^il^^Aθ^*^’^ |*R* > and |*L, l_B_*> = *e^−il^^Bθ^*^’^ |*L* > with topological charges *l_A_* and *l_B_*, respectively, where |*R* > = [1 − *i*]^T^ and |*L* > = [1 *i*]^T^ were Jones vectors of the RCP and LCP, and *θ*’ was the azimuth angle. The two sets of nanoslits A and B were arranged alternatively in the *y*-direction, and for a single set of slits, the spacings of adjacent slits in the *x*-direction and *y*-direction were D and 2D, respectively. The combination of the two sets of nanoslits forms the spatially multiplexing metasurface with a slit-to-slit spacing D in both the *x*-and *y*-directions. Owing to the fact that the converged vortex beams of RCP (or LCP) can be generated by the set of nanoslits A (or B), under the illumination of LCP (or RCP), the metasurface can independently manipulate the two eigenstates of vortices |*R, l_A_*> and |*L, l_B_*>, under LP incident light which contains LCP and RCP components. Figure 1b shows a magnified view of a single nanoslit, which was designed in an Au film with a thickness of H = 200 nm on glass substrate. The length and width of each nanoslit were 250 nm and 80 nm, respectively. The orientation angle of the longer side of the slit is *φ* with respect to *x*-axis.

A single slit can be considered as an anisotropic polarizer, and the coefficients for the transmitted light field of the nanoslit can be described by Jones matrix when it rotates angle *φ* around the *z*-axis:

(1) $$J(\varphi )=R(-\varphi )\left[\begin{array}{cc} t_{x} & 0\\ 0 & t_{y}e^{i\alpha } \end{array}\right]R(\varphi )$$

where *t_x_*and *t_y_* are the amplitudes of transmission electric fields along the two major axes of the nanoslit as the polarizer, *α* is the phase delay between the electric fields and R(φ) is the rotation matrix. The transmission coefficients of slit to incident light field written as [24]:

(2) $$J(\varphi )=\left[\begin{array}{cc} t_{x}\cos^{2}\varphi +t_{y}\sin^{2}\varphi e^{i\alpha } & (t_{x}-t_{y}e^{i\alpha })\cos\varphi \sin\varphi \\ (t_{x}-t_{y}e^{i\alpha })\cos\varphi \sin\varphi & t_{x}\sin^{2}\varphi +t_{y}\cos^{2}\varphi e^{i\alpha } \end{array}\right].$$

For the slit in this paper, it can be taken as a polarizer, so *t_x_* = 1 and *t_y_* = 0, the transmission matrix of unitary cell is derived as:

(3) $$J_{c}(\varphi )=\left[\begin{array}{cc} \cos^{2}\varphi & \cos\varphi \sin\varphi \\ \cos\varphi \sin\varphi & \sin^{2}\varphi \end{array}\right].$$

Given a CP incident wave $E_{in}^{\sigma }=(1/\sqrt{2})[1\;i\sigma {]}^{T}$, where *σ* = ±1 represents LCP or RCP light, respectively. Following $E_{T}^{\sigma }=J_{c}(\varphi )\cdot E_{in}^{\sigma }$, the transmitted light field of a single slit can be expressed as:

(4) $$E_{T}^{\sigma }=[E_{in}^{\sigma }+E^{-\sigma }\exp(i2\sigma \varphi )]/2\sqrt{2},$$

where $E^{\sigma }$ and $E^{-\sigma }$ denote the incident polarization helicity of *σ* and −*σ*, respectively. The value of spin angular momentum iσℏ depends on the helicity of the CP. Equation (4) indicates that under illumination of CP light, the transmitted light field $E_{T}^{\sigma }$ was divided into two components: One was the incident spin component that has the same helicity as the incident CP light, and the other was the converted spin component that has opposite helicity to the incident light. The converted spin component acquires the geometric (Pancharatnam–Berry (PB)) phase shift of 2*σφ*, which depends on the orientation angle *φ* of the nanoslits as well as the CP helicity *σ*. The designed PB phase profile of the metasurface was realized by rotating the nanoslits with certain angles in a spatially inhomogeneous array. In order to generate a focused trefoil and cinquefoil vector light field, the two sets of nanoslits A and B were designed to carry the corresponding metalens phase *ϕ_lens_*(*x, y*) and vortex phase *ϕ_v,q_*(*x, y*) by varying orientations of the nanoslits at different positions. Therefore, the superposition of these two phases for each nanoslit set constitutes the phase profile, i.e., *ϕ_p_*(*x, y*) = *ϕ_lens_*(*x, y*) + *ϕ_v,q_*(*x, y*), and it was encoded into the corresponding orientation angle of the nanoslit *φ*(*x, y*) = *φ_lens_*(*x, y*) + *φ_v,q_*(*x, y*) in considering the relation of PB phase *ϕ* = 2*σφ* with *φ*.

Next, we analyzed the vortex and metalens phase encoding of the two sets of nanoslits A and B. To yield a optical vortex, the required orientation angle *φ_v,q_*(*x*, *y*) of a nanoslit at the position of (*x*, *y*) is written as:

(5) $$\varphi_{v,q}(x,y)=\varphi_{0}+q\theta ,$$

where is *φ*_0_ the initial orientation angle at azimuth *θ* = 0 and *q* is the rotation order of the slits. *θ* = *θ*_0_ + (arctan(*y*/*x*)) is the azimuth of the slit, and *θ*_0_ is a constant. When *θ* varies from 0 to 2*π*, the orientation angle of the slit changes 2*πq*, enabling the topological charge of the vortex *l* = 2*σq*. The vortex phase can be obtained by rotating the nanoslit with *ϕ_v,l_*(*x*, *y*) = 2*σφ_v,q_*(*x*, *y*). Furthermore, to achieve the PB phase distribution of the metalens *ϕ_lens,σ_*(*x*, *y*) = 2*σφ_lens,σ_*(*x*, *y*), the orientation angle *φ_lens,σ_*(*x*, *y*) for the nanoslit at (*x*, *y*) is expressed as:

(6) $$\varphi_{lens,\sigma }(x,y)=\frac{\sigma \pi }{\lambda }(\sqrt{x^{2}+y^{2}+f^{2}}-f),$$

where λ is the wavelength, and *f* is the focal length of the metalens. We design the set A of nanoslits with the metalens and vortex phases to generate the focused RCP vortex beam converted from the LCP incident light, and the set B with phases to generate the focused LCP vortex beam converted from the RCP incident light; correspondingly, for set A and set B, the orientation angle of their nanoslits are written as:

(7) $$\varphi_{A}=\varphi_{0A}-\frac{\pi }{\lambda }(\sqrt{x^{2}+y^{2}+f^{2}}-f)+q_{A}\left[\theta_{0}+\arctan(y/x)\right],$$

(8) $$\varphi_{B}=\varphi_{0B}+\frac{\pi }{\lambda }(\sqrt{x^{2}+y^{2}+f^{2}}-f)+q_{B}\left[\theta_{0}+\arctan(y/x)\right],$$

where *φ*_0A_ and *φ*_0B_ are the initial orientation angles of two sets of nanoslits A and B, respectively, *q*_A_ and *q*_B_ are referred to as the rotation orders of set A and set B, respectively, and *θ*_0_ = π/2 for *x* < 0, and else *θ*_0_ = 0. Here we note that the orientation angles for metalens phases in Equations (7) and (8) are in opposite signs, indicating that set A and set B of the nanoslits focuses the RCP field and the LCP field, respectively; correspondingly, they diverge the fields of LCP and RCP, respectively.

To produce a converged trefoil or cinquefoil vector light field, the LP light $E_{in}^{\left(lp\right)}$ which contains light of LCP and RCP needs to be employed for illumination, so that set A generates and focuses the RCP vortex |*R, l_A_*> as the spin component converted from the LCP in the illuminating light, while set B generates and focuses the LCP vortex |*L, l_B_*> as the spin component converted from the RCP in the illuminating light. The LP light $E_{in}^{\left(lp\right)}$ for illumination can simply expressed as:

(9) $$E_{in}^{\left(lp\right)}=\sum_{\sigma =-1\left(\sigma \neq 0\right)}^{1}\left[\begin{array}{c} 1\\ i\sigma \end{array}\right]e^{-i\sigma \delta_{0}}=\sum_{\sigma =-1\left(\sigma \neq 0\right)}^{1}E_{in}^{\sigma }e^{-i\sigma \delta_{0}}\;,$$

where the coefficient $1/\sqrt{2}$ is omitted for simplicity, *δ*_0_ is the relative phase difference between the two CPs, and it determines direction of the linear polarization. The generic analytical calculation can be realized based on Equation (4) together with Equation (9) and on the Huygens–Fresnel principle of surface plasmon polaritons (SPP) excited by nanoslit [25]. Thereby, as shown in Figure 1c, at point *M* (*x*’*, y*’) near the center of the observation plane *x*’*oy*’, the wave field *E_tot_*(*x*’*, y*’) is written as:

(10) $$\begin{array}{ll} E_{tot}\left(x^{\prime },y^{\prime }\right) & =\sum_{\sigma =-1\left(\sigma \neq 0\right)}^{1}-\frac{i}{\sqrt{\lambda }}\iint_{S}\{\;\left[\begin{array}{c} 1\\ i\sigma \end{array}\right]e^{-i\sigma \delta_{0}}+\left[\begin{array}{c} 1\\ -i\sigma \end{array}\right]e^{i\sigma \left(\delta_{0}+2\varphi_{A}\right)}\}\;e^{ik\rho }\frac{1}{\rho }dxdy\\ & +\sum_{\sigma =-1\left(\sigma \neq 0\right)}^{1}-\frac{i}{\sqrt{\lambda }}\iint_{S}\{\;\left[\begin{array}{c} 1\\ i\sigma \end{array}\right]e^{-i\sigma \delta_{0}}+\left[\begin{array}{c} 1\\ -i\sigma \end{array}\right]e^{i\sigma \left(\delta_{0}+2\varphi_{B}\right)}\}\;e^{ik\rho }\frac{1}{\rho }dxdy \end{array}$$

where *k = 2π/λ* is the free-space wave vector, $\rho =\sqrt{{\left(x^{\prime }-x\right)}^{2}+{\left(y^{\prime }-y\right)}^{2}+f^{2}}$ is the distance from *m* to *M*, *m*(*x*, *y*) is the position of the nanoslit and the integral is over the area *S* of the metasurface. The two summations in Equation (10) are for nanoslit set A and set B, respectively. It can be seen that each of the CP components, i.e., the LCP or the RCP, in the LP illuminating light produces two light fields of the incident spin and converted spin components by each of two nanoslit sets, i.e., set A or set B; therefore, the wave field produced by a spatially multiplexing metasurface contains eight components. Four of these components were produced by the set of nanoslit A, including two incident spin components of LCP and RCP corresponding to that in the illuminating light, and two the converted spin components of RCP and LCP that carries the PB phases *e^i2φ^*^A^ and *e*^−*-i2φ*^^A^ stemmed from the rotating orientations of the set of nanoslit A, respectively. The other four components were produced by the set of nanoslit B are the incident spin components LCP and RCP, as well as the converted spin components RCP and LCP with the PB phases *e^i2φ^*^B^ and *e*^−*i2φ*^^B^. ­Among these components, the four incident spin components produced by both nanoslit set A and set B were not controlled by the designed PB phases, but they transmit through the metasurface and diffract onto observation plane as almost negligible background. In order to further reduce the influence of this background on the desired fields, we removed part of the nanoslits in the center area of the metasurface, which is shown to greatly reduce the adverse background intensity and improve the quality of the generated focused vector field. Although the exclusion of the slits in central region may cause the loss of energy and lower the transmission coefficient of the overall system, this eliminates harmful influence on the focused vector beam from incident spin component of these slits, and avoids their intense contribution to background due to the diffraction right along the propagation direction. Thereby, the advantage of this metasurface design lies in that it can greatly improve the quality of the generated vector beams, especially important for highly demanding light field regulation. Whereas in our design, the set of nanoslits A produced the focused vortex beams of RCP under illumination of the LP light, the corresponding metalens phase imposed to the set of nanoslits A by rotating the orientation angle of the slit focuses field of RCP component on the observation plane, and diverged field of LCP component which was actually taken as an even weaker background. A similar mechanism was true for nanoslit set B to produce the focused vortex beams of LCP. Consequently, when a plane wave of LP light illuminates the spatially multiplexing metasurface, the effective components of the light field were the focused converted spin component of RCP generated by the nanoslit set A and that of LCP by the nanoslit set B. From Equation (10), the focused vector field can be equivalently written as:

(11) $$E_{tot}(x^{\prime },y^{\prime })=-\frac{i}{\sqrt{\lambda }}[\iint_{S}e^{i\left(2\varphi_{A}+k\rho +\delta_{0}\right)}\frac{1}{\rho }dxdy]|R>-\frac{i}{\sqrt{\lambda }}[\iint_{S}e^{-i\left(2\varphi_{B}-k\rho +\delta_{0}\right)}\frac{1}{\rho }dxdy]|L>.$$

With *x’* << *x*, *y’* << *y* into account, the distance *ρ* is approximated:

(12) $$\rho =\sqrt{x^{2}+y^{2}+f^{2}-2xx^{\prime }-2yy^{\prime }}.$$

Substituting Equations (7), (8) and (12) into Equation (11), and with some simple calculations and reasonable approximation, we obtain:

(13) $$\begin{array}{ll} E_{tot}(x^{\prime },y^{\prime }) & =-\frac{i}{\sqrt{\lambda }}e^{i\left(2\varphi_{0A}+kf+\delta_{0}\right)}[\iint_{S}e^{il_{A}\theta }e^{-ik(xx^{\prime }+yy^{\prime })/f}\frac{1}{\rho }dxdy]|R>\\ & -\frac{i}{\sqrt{\lambda }}e^{-i\left(2\varphi_{0B}-kf+\delta_{0}\right)}[\iint_{S}e^{-il_{B}\theta }e^{-ik(xx{}^{\prime }+yy{}^{\prime })/f}\frac{1}{\rho }dxdy]\;|L> \end{array}$$

further calculations of the above equation may depend on the complexity of integral area *S*; we look at the case when *S* is a circular region of radius *R*, and then the aperture for the integral function is expressed as the circle function circ(r/R). By transforming Cartesian coordinates (*x, y*) into polar coordinates (*r*, *θ*) as the variables in the integrals in Equation (13), the light field *E* (*r’, θ’*) on observation plane can be written:

(14) $$\begin{array}{ll} E(r^{\prime },\theta^{\prime }) & =C[{\left(-i\right)}^{l_{A}+1}kf^{-1}e^{i(2\varphi_{0A}+\delta_{0})}e^{il_{A}\theta {}^{\prime }}\int_{0}^{R}J_{l_{A}}(krr{}^{\prime }/f)rdr|R>\\ & +C[{\left(-i\right)}^{l_{B}+1}kf^{-1}e^{-i(2\varphi_{0B}+\delta_{0})}e^{-il_{B}\theta {}^{\prime }}\int_{0}^{R}J_{l_{B}}(krr{}^{\prime }/f)rdr]|L> \end{array}$$

where $C=-ie^{ikf}/\sqrt{\lambda \left(R^{2}+f^{2}\right)}$ and *ρ* in the denominator of Equation (13) can be written as $\left|\rho \right|=\sqrt{\left(R^{2}+f^{2}\right)}$, (*r’, θ’*) are polar coordinates in the observation plane and *J_l_*(*x*) is the Bessel function of the *l*th order and first kind. Further derivation of Equation (14) gives the following expression,

(15) $$E\left(r{}^{\prime },\theta {}^{\prime }\right)=C[\psi_{1circ}(r^{\prime })e^{i(2\varphi_{0A}+\delta_{0})}e^{il_{A}\theta {}^{\prime }}|R>+\;\psi_{2circ}(r^{\prime })e^{-i(2\varphi_{0B}+\delta_{0})}e^{-il_{B}\theta {}^{\prime }}|L>],$$

where

(16) ψ1circ(r′)=(−i)lA+1(lA+2)lA!(kR2f)(kRr′2f)lAF12[lA+22,lA+42,lA+1;−(kRr′2f)2],

(17) ψ2circ(r′)=(−i)lB+1(lB+2)lB!(kR2f)(kRr′2f)lBF12[lB+22,lB+42,lB+1;−(kRr′2f)2].

${}_{1}F_{2}\left(a,b,c;x\right)$ is a hypergeometric function [26]. Equation (15) together with Equations (16) and (17) indicates that *E* (*r*’*, θ*’) are the desired superposition of two eigen vector vortices of *e^il^^Aθ^*^’^ |*R* > and *e^−il^^Bθ^*^’^ |*L* >, by which the vector fields were obtained. While phase factors *e^i2φ^*^0A^ and *e^−i2φ^*^0B^ determines the initial polarization states of the vector beam, the function *ψ*_1*circ*_(*r*’) and *ψ*_2*circ*_(*r*’) denote the doughnut-like radial distributions of the intensity, and they were focused at the around the center of observation plane, where cores of focused eigen vortices coincide. When the aperture for the integral area takes different shapes, such as the familiar shapes of annular and rectangular apertures, slightly affect the doughnut profiles of the intensity distribution of the eigen vortices may change to some degree, but essential the characteristics of the vortices will not be influenced much. Therefore, without losing generality, the focused vector field in Equation (15) produced by the spatially multiplexing metasurface can be written as:

(18) $$E(r{}^{\prime },\theta {}^{\prime })=C[\psi_{1}(r^{\prime },\theta^{\prime })e^{i(2\varphi_{0A}+\delta_{0})}e^{il_{A}\theta {}^{\prime }}|R>+\;\psi_{2}(r^{\prime },\theta^{\prime })e^{-i(2\varphi_{0B}+\delta_{0})}e^{-il_{B}\theta {}^{\prime }}|L>]$$

where *ψ*_1_(*r*’, *θ*’) and *ψ*_2_(*r*’, *θ*’) are the generalized doughnut profiles of the focused vortices, they depend on the area on which nanoslits of the metasurface are arranged, and generally they are functions of (*r*’, *θ*’). In Equation (18) it is seen that *E* (*r*’, *θ*’) is the superposition of two vector vortices |*R, l_A_*> and |*L, l_B_*>. The focused trefoil vector field was the superposition of |*R*, 1> and |*L*, 2>, and the focused cinquefoil vector field was the superposition of |*R*, 3> and |*L*, 2>. Under illumination of LP light, when *l_A_* = 1 and *l_B_* = 2, the two eigen vortices |*R*, 1> and |*L*, 2> were achieved, respectively. Consequently, their superposition creates the focused trefoil vector field. When *l_A_* = 3 and *l_B_* = 2, the two eigen vortices were |*R*, 3> and |*L*, 2>, and the focused cinquefoil vector field were created. Similarly, when *l_A_* = −1 or −3, *l_B_* = −2, the eigen vortices with the topological charge reversed (i.e., |*R*, −1> and |*L*, −2>, |*R*, −3> and |*L*, −2>) were obtained, and the focused trefoil π-vector field and focused cinquefoil π-vector field were created.

## 3. Results and Discussions

Based on the above theory, we conducted theoretical simulation according to Equation (13), and obtained the expected result. Then, the transmitted fields of the metasurfaces are simulated with the finite-difference time domain (FDTD, Lumerical FDTD-Solution) method. Samples S_1_ and S_2_ of metasurfaces were designed to generate trefoil vector field and trefoil π-vector field respectively. Samples S_3_ and S_4_ of metasurfaces were designed to generate cinquefoil vector field and cinquefoil π-vector field respectively. By changing direction of the incident linear polarization, the vector fields with different polarization states can be generated. In addition, in order to optimize the simulation results of the vector fields, the spacing of nanoslit in the metasurface was slightly adjusted with different LP light illuminations, and the range of adjustment was within 10 nm. The wavelength of the incident light was set to 632.8 nm. The intensities of the vector wave field were observed on the focal plane of *f* = 10 μm from the gold film.

**Figure 2:**
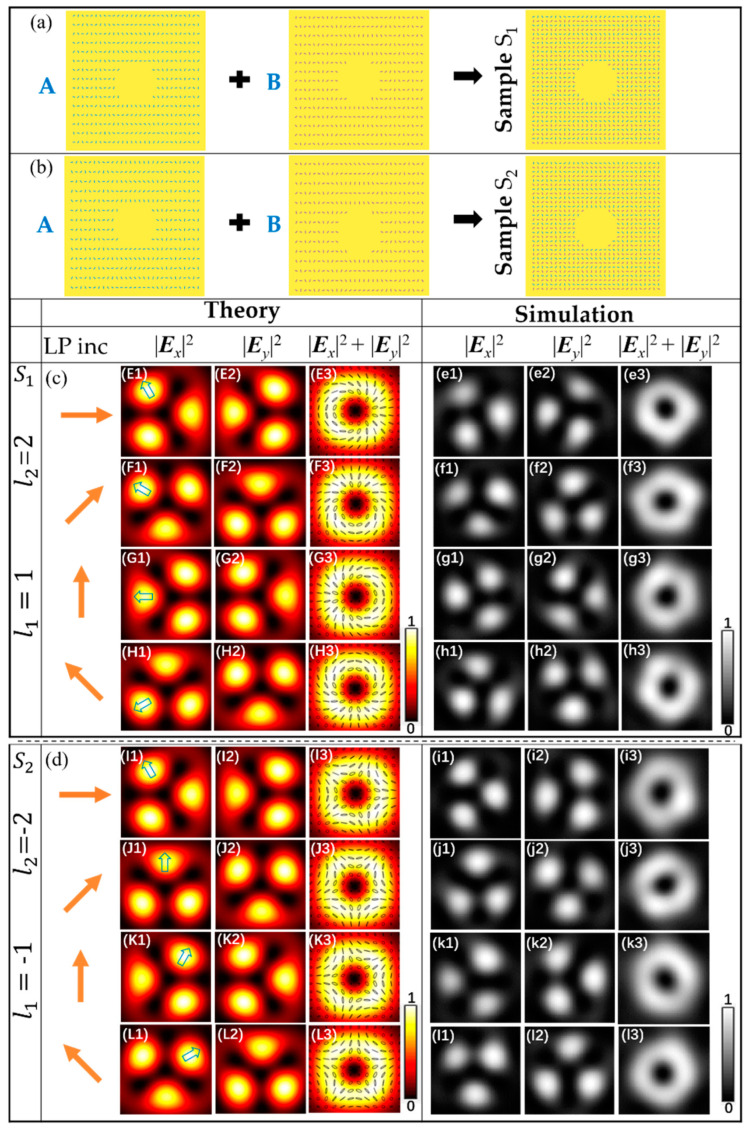
Theoretical and simulated intensity profiles of the vector beam for the samples S_1_ and S_2_ under LP illuminations. (**a**) schematic of the structure of sample S_1_. (**b**) schematic of the structure of sample S_2_. Samples S_1_ and S_2_ with parameter (*l_A_, l_B_*) = (1, 2) and (−1, −2) are shown in the first column of (**c**) and (**d**), respectively. (**c**) patterns from the top to bottom rows are different polarized trefoil vector beam under the illumination of different LP light, respectively. (**d**) patterns from the top to bottom rows are corresponding trefoil π-vector beam, respectively. The schematics of the incident light and the intensity patterns of |*E_x_*|^2^, |*E_y_*|^2^, and |*E_x_*|^2^ + |*E_y_*|^2^ are shown from left to right.

The intensity patterns of the transmitted fields by theoretical calculation and FDTD simulation for samples S_1_ and S_2_ are shown in Figure 2. The parameters of the two samples were (*l*_A_, *l*_B_)_S1_ = (1, 2) and (*l*_A_, *l*_B_)_S2_ = (−1, −2), respectively, and the initial nanoslit angles *φ*_0A_ = *π*/4 and *φ*_0B_ = *π*/2. Figure 2 shows the intensity patterns and polarization distribution of the vector field with the incident polarization directions at 0°, 45°, 90° and 135° with respect to the *x*-axis. From the Figure 2, we see that the theoretical results are well consistent with those of FDTD simulation. The results by theoretical calculation and FDTD simulation for sample S_1_ are shown in Figure 2E1–H3,a1–h3, respectively, and represent the superposition of |*R*, 1> + |*L*, 2>. The patterns in Figure 2E1–3 in the first row show the results, under the illumination of horizontally polarized light. Figure 2E1,2 show the intensity pattern of the components |*E_x_*|^2^ and |*E_y_*|^2^, respectively, we can see that the intensity patterns of both |*E_x_*|^2^ and |*E_y_*|^2^ have the patterns of three lobes with various orientations divided by the dark lines, similar to the trefoil. Figure 2E3 shows the theoretically normalized intensity |*E_x_*|^2^ + |*E_y_*|^2^ of the vector field, with the polarization state of theoretical calculation overlaid on the patterns, and it can be seen that the spatially varying polarization states include linear, elliptical and circular polarizations. The direction of polarization is also constantly changing with different spatial points. When changing direction of the incident linear polarization, the change of *δ*_0_ leads to rotation of polarization at a fixed point in real space. This rotation can be reflected more intuitively in the rotation of beam patterns, we marked a lobe in each pattern with hollow blue arrow, the angle *τ* of pattern can be obtained from the marked lobe and the vector beam pattern rotates with *δ*_0_ by Δτ = −*δ*_0_/(*l*_A_ + *l*_B_) [27]. Figure 2F1–3 in the second row show the results from the sample S_1_ with the incident polarization directions at 45° with respect to the *x*-axis, with respect to the phase shift *δ*_0_ = *π*/2. From Figure 2F1,2, we see that the marked lobe rotates angle 30° counterclockwise compared to the horizontally polarized light illumination. The distribution of polarization states also varies as shown in Figure 2F3. For LP with the polarization directions is 90° and 135° illumination, the marked lobe rotates angle 60° and 90° counterclockwise, respectively, compared to the horizontally polarized light illumination, and the distribution of polarization states will also change accordingly, corresponding to Figure 2G1–3,H1–3, respectively. The theoretical results and simulation results for sample S_2_ are shown in Figure 2I1–L3,i1–l3, respectively, and represent the superposition of |*R*, −1> + |*L*, −2>. Under different LP light illuminations, the trefoil *π*-vector fields can be obtained, and the marked lobe rotates clockwise.

**Figure 3:**
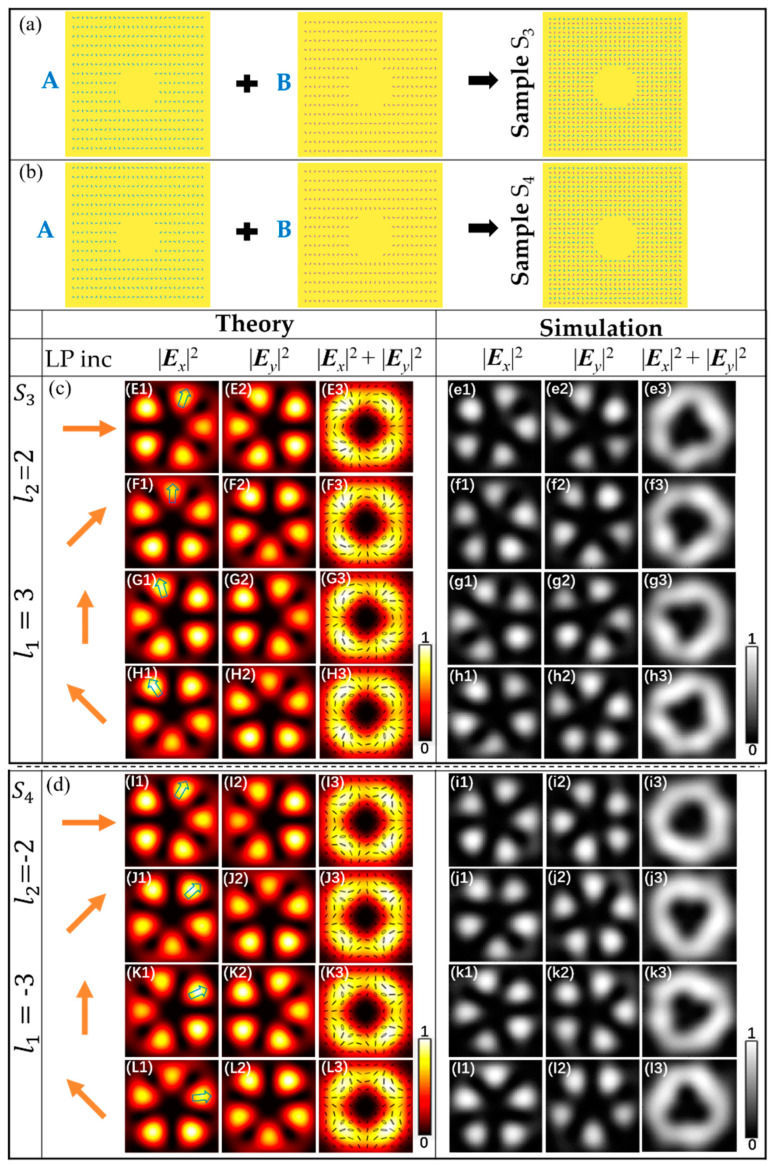
Theoretical and simulated intensity profiles of the vector beam for the samples S_3_ and S_4_ under LP illuminations. (**a**) schematic of the structure of sample S_3_. (**b**) schematic of the structure of sample S_4_. The two samples S_3_ and S_4_ with parameter (*l_A_, l_B_*) = (3, 2) and (−3, −2) are shown in the first column of (**c**) and (**d**), respectively. (**c**) and (**d**) patterns from the top to bottom rows are different polarized cinquefoil vector beam and cinquefoil π-vector beam under LP light with different polarization directions, respectively. The schematics of the incident light and the intensity pattern of |*E_x_*|^2^, |*E_y_*|^2^, and |*E_x_*|^2^ + |*E_y_*|^2^ are shown from left to right.

For sample S_3_ and S_4_, the parameters were (*l*_A_, *l*_B_)_S3_ = (3, 2) and (*l*_A_, *l*_B_)_S4_ = (−3, −2), respectively, and the initial nanoslit angles *φ*_0A_ = 3*π*/4 and *φ*_0B_ = *π*/2. The theoretical results and simulation results of sample S_3_ and sample S_4_ are shown in Figure 3E1–H3,e1–h3; Figure 3I1–L3,i1–l3, respectively, for the superposition of |*R*, 3> + |*L*, 2> and |*R*, −3> + |*L*, −2>. The theoretical and simulation results showed good agreement. Under the illumination of horizontally polarized light of the samples S_3_, the intensity patterns are shown in Figure 3E1–3. It can be seen from Figure 3E1,2 that the components |*E_x_*|^2^ and |*E_y_*|^2^ have the patterns of five lobes with various orientations divided by the dark lines, similar to the cinquefoil. Linear, elliptic, and circular polarization states are simultaneously contained in the vector field, and the direction of polarization are constantly changing with different spatial points, as shown in Figure 3E3. Under the illumination of LP with the polarization directions is 45°, the labeled lobe rotates angle 18° counterclockwise compared to the horizontally polarized light illumination, and the distribution of the polarization states of the vector field also varies, as shown in Figure 3F1–3. Figure 3G1–3,H1–3 in the third and fourth row show the results from the sample S_3_ for LP with the polarization directions is 90° and 135°, respectively. It can be seen that the labeled lobe rotates angle 36° and 54° counterclockwise, respectively, compared to the horizontally polarized light illumination. For sample S_4_, under the illumination of LP light, the cinquefoil π-vector fields can be obtained. By changing the polarization direction of LP light, the labeled lobe rotates clockwise.

## 4. Conclusions

In conclusion, we propose spatially multiplexing of metasurface comprising two sets of nanoslit to generate focused trefoil and cinquefoil vector field by adjusting the PB phase. The vector light fields with different polarization states are obtained by changing direction of the incident linear polarization. In addition, the generated fields contain the spatially varying polarization states include linear, elliptical and circular polarizations, and the direction of polarization changes continuously with spatial position. Theoretical derivation is presented, and numerical simulations are carried out. The results demonstrate that the multiplexing of metasurface can be used to manipulate the optical field flexibly and to generate feasibly focused trefoil and cinquefoil vector field, which will be great significance to broaden the application area of metasurfaces and to explore the nanoscale topological optical field.

## Data Availability

Data is contained within the article.
